# Immune cell populations and induced immune responses at admission in patients hospitalized with vaccine breakthrough SARS-CoV-2 infections

**DOI:** 10.3389/fimmu.2024.1360843

**Published:** 2024-06-05

**Authors:** Adin Sejdic, Hans Jakob Hartling, Jon Gitz Holler, Lars Klingen Gjærde, Birgitte Lindegaard, Arnold Matovu Dungu, Filip Gnesin, Maria Elizabeth Engel Møller, Rebecca Svanberg Teglgaard, Carsten Utoft Niemann, Patrick Terrence Brooks, Charlotte Sværke Jørgensen, Kristina Træholt Franck, Thea K. Fischer, Hanne Vibeke Marquart, Zitta Barrella Harboe, Sisse Rye Ostrowski

**Affiliations:** ^1^ Department of Pulmonary and Infectious Diseases, Copenhagen University Hospital – North Zealand, Hillerød, Denmark; ^2^ Department of Clinical Medicine, Faculty of Health and Medical Sciences, University of Copenhagen, Copenhagen, Denmark; ^3^ Department of Clinical Immunology, Copenhagen University Hospital - Rigshospitalet, Copenhagen, Denmark; ^4^ Department of Hematology, Copenhagen University Hospital - Rigshospitalet, Copenhagen, Denmark; ^5^ Department of Cardiology, Copenhagen University Hospital – North Zealand, Hillerød, Denmark; ^6^ Virus & Microbiological Special Diagnostics, Statens Serum Institut, Copenhagen, Denmark; ^7^ Department of Clinical Research, Copenhagen University Hospital – North Zealand, Hillerød, Denmark

**Keywords:** immune cell populations, inflammation, vaccine breakthrough infection, mRNA vaccine against SARS-CoV2, cytokines

## Abstract

**Background:**

Vaccine breakthrough SARS-CoV-2 infections are common and of clinical and public health concern. However, little is known about the immunological characteristics of patients hospitalized due to these infections. We aimed to investigate and compare immune cell subpopulations and induced immune responses in vaccinated and non-vaccinated patients hospitalized with severe COVID-19.

**Methods:**

A nested case-control study on adults (≥ 18 years) who received at least two doses of a mRNA-COVID-19 vaccine and were hospitalized with SARS-CoV-2 breakthrough infections and severe COVID-19 between January 7, 2021, and February 1, 2022, were eligible for inclusion. Age- and sex-matched non-vaccinated controls were identified. Immunophenotyping was performed using a custom-designed 10-color flow cytometry prefabricated freeze-dried antibody panel (DuraClone, Beckman Coulter (BC), Brea, Calif). TruCulture (Myriad RBM, Austin, USA) was used to assess induced immune response in whole blood, revealing different critical signaling pathways as a proxy for immune function. All samples were obtained within 48 hours of admission.

**Results:**

In total, 20 hospitalized patients with severe COVID-19 and a breakthrough SARS-CoV-2 infection were included, ten vaccinated and ten non-vaccinated patients. Vaccinated patients had lower concentrations of CD19 B cells (p = 0.035), naïve CD4 T cells (p = 0.015), a higher proportion of γδ1 T cells (p = 0.019), and higher unstimulated immune cell release of IL-10 (p = 0.015).

**Conclusion:**

We observed immunological differences between vaccinated and non-vaccinated patients hospitalized due to severe COVID-19 that indicate that vaccinated patients had lower B cell concentrations, lower concentrations of CD4 naïve T cells, a skewed gamma-delta V1/V2 ratio, and an exaggerated IL-10 response at admission. These results could indicate a suboptimal immune response involved in SARS-CoV-2 breakthrough infections that cause severe COVID-19 in vaccinated adults. However, the sample size was small, and further research is needed to confirm these results.

## Introduction

Coronavirus disease 2019 (COVID-19) is a respiratory infection caused by the β-coronavirus severe acute respiratory syndrome coronavirus 2 (SARS-CoV-2) and the cause of the current pandemic (1). By December 2023, nearly 700 million cases have been registered, including more than 6.9 million deaths (2). Since the introduction of mass vaccination in December 2020, overall mortality and morbidity have been dramatically reduced (3). However, due to the emergence of new SARS-CoV-2 variants that bypass vaccine-induced immunological protection, the risk of severe COVID-19 disease requiring hospital admission in vaccinated patients remains a clinical and public health concern (4–6).

The decline in protective immunity, attributed to waning levels of antibodies and T-cell immunity post-vaccination, and the emergence of novel SARS-CoV-2 variants, constitute the two main factors contributing to the substantial increase in vaccine breakthrough SARS-CoV-2 infections worldwide (5–8). Even if vaccination has proven to be associated with a significantly reduced risk of severe outcomes and death in individuals of all ages (9–13), SARS-CoV-2 breakthrough infections remain a significant cause of hospitalization (14). Exploring the immunological characteristics of breakthrough SARS-CoV-2 infections is crucial for understanding the dynamics of vaccine efficacy over time, the potential immune responses that may impact disease severity, and, eventually, optimizing vaccination strategies to ensure the protection of individuals at the highest risk of severe outcomes.

We aimed to investigate and compare immune cell subpopulations and the induced immune responses comparing vaccinated and non-vaccinated patients hospitalized due to severe COVID-19. We hypothesized that vaccinated patients admitted with severe COVID-19 have a non-favorable immune profile, which could indicate immune impairment compared to non-vaccinated patients. We also aimed to identify overall differences in the immunologic signatures of SARS-CoV-2 breakthrough infections, aiming to provide a foundation for subsequent investigations.

## Materials and methods

### Study design

We conducted an observational nested case-control study with the main objective of describing the immunologic differences at hospital admission between vaccinated (cases) and non-vaccinated (controls) adult (≥ 18 years) patients hospitalized with severe COVID-19. In addition, we retrospectively assessed clinical outcomes for the whole hospitalization period and evaluated mortality 90 days after admission. The characteristics of the complete COVIMUN cohort have been described elsewhere (15).

Patients admitted to Copenhagen University Hospital - North Zealand, Hillerød, Denmark, were screened for inclusion in the study between January 7, 2021, and February 1, 2022. During the study period, all patients admitted to the hospital due to respiratory symptoms were screened for SARS-CoV-2 infection by oropharyngeal swabs or tracheal aspirates, using reverse transcriptase-polymerase chain reaction (RT-PCR) at the time of admission, to comply with hospital infection control policies. Patients’ eligibility for inclusion in the study was based on two inclusion criteria (1): a positive diagnostic SARS-CoV-2 RT-PCR at admission and (2) a history of vaccination with a minimum of 2 doses 14 days before admission. SARS-CoV-2 variants (Delta or Omicron) were recorded when available. Data regarding vaccination status was retrieved from the Danish Vaccination Database (DDV) (16). Patients with a history of only one vaccine dose or <14 days between the second (or third) vaccination and hospitalization were excluded. All patients in the vaccinated group received the mRNA Pfizer/Biontech (Comirnaty, BNT162b2) vaccine. In addition, patients from the COVIMUN cohort hospitalized with RT-PCR-confirmed SARS-CoV-2 infection and no confirmed vaccination history before admission were used to identify age- and sex-matched controls. The research received approval from the Danish Ethics Committee (H-20026502) and the Danish Data Protection Agency (P-2020–426), adhering to the principles of the Declaration of Helsinki. Written informed consent was obtained from all participants in the study.

### Variables and outcomes

Clinical, demographic, and outcome variables were retrospectively extracted from the patient’s electronic medical journals. We retrieved information on age, sex, comorbidities, body mass index (BMI), immunosuppressive treatment, immunodeficiency disorders, oxygen treatment during admission, vital signs, intensive care unit (ICU) admission, in-hospital and 90-day mortality. Vital signs at admission were used to calculate the Early Warning Score (EWS) to assess patient clinical deterioration (17). Immunosuppressive treatment was defined based on our previous research (18) as the use of (1) corticosteroid treatment exceeding a prednisolone-equivalent dose of 20 mg daily ≥ 14 days at the time of admission, (2) monoclonal antibodies interfering with the immune system, (3) small-molecule immunosuppressive drugs, or (4) antineoplastic agents. Disease severity was defined based on the peak oxygen supplementation treatment needed during admission, as in our previous study (18). Severe disease was defined as treatment with a high-flow nasal cannula (HFNC), invasive mechanical ventilation, or non-invasive mechanical ventilation (NIV) during admission. All other oxygen treatments were defined as mild disease (nasal cannula or oxygen mask). All TruCulture and DuraClone samples were obtained within 48 hours of admission.

### Immunophenotyping by flow cytometry

Immunophenotyping was performed using a custom-designed 10-color flow cytometry prefabricated freeze-dried antibody panel (DuraClone, Beckman Coulter (BC), Brea, Calif) specially designed for evaluation of leukocyte subsets in primary and secondary immunodeficient patients (19). The first tube contained beads used to calculate absolute concentrations of lineage populations and further calculate the concentrations of all other subpopulations in the other tubes. Peripheral blood was obtained in EDTA tubes and processed within 24 hours according to the manufacturer’s instructions. In summary, whole blood was stained for 15 min at room temperature, then subjected to red blood cell lysis (EasyLyse, BC), washed, and analyzed on a Beckman Coulter Navios Ex flow cytometer. For staining of intracellular markers (Foxp3 and Helios), cells were permeabilized and fixated using Perfix Buffers 1–3 (BC). Data was analyzed using Beckman Coulter’s Kaluza Analysis 2.1 software. The leukocyte subpopulations were defined *a priori*, using serial gating strategies. The defined subpopulations, including T cells, B cells, differentiation stages, activation state, and exhaustion, were specified as absolute concentrations (x10^9^/L) or percentages of the parent gate (%), depending on the variable (19). Normal ranges from routine analyses of absolute counts of specific cells were used as normal reference material. This included concentrations of leukocytes, neutrophils, lymphocytes, monocytes, T-cells (CD4 and CD8), B-cells, and NK cells.

### TruCulture: induced immune response analysis

TruCulture (Myriad RBM, Austin, USA) was used to assess the induced immune response in whole-blood, i.e., revealing different critical signaling pathways, as a proxy for immune function (20, 21). In brief, heparinized whole-blood samples were added to pre-coated (stimulated or unstimulated) TruCulture tubes within 60 minutes according to the manufacture’s recommendation. The TruCulture tubes were incubated in a digital dry block heater at 37**°**C for 22 hours. After that, supernatants were collected and frozen at -80°C until analysis. We assessed four different stimuli and an unstimulated blank: Lipopolysaccharide (LPS, Toll-Like-Receptor (TLR)4 ligand), Resiquimod (R848, TLR7/8 ligand), Polyinosinic: polycytidylic acid (Poly I:C, TLR3 ligand), anti-CD3 anti-CD-28 (CD3/CD28, T-cell stimulation) and a blank (NULL, cell culture medium without stimulants).

We investigated nine different cytokines in each supernatant using a Luminex 200 instrument (LX200, R&D Systems, BIO-Techne LTD, Abingdon, UK): interleukin (IL) -1β, IL-6, IL-8, IL-10, IL-12, IL-17A, interferon (IFN)-α, IFN-γ and tumor necrosis factor (TNF)-α. The selection of cytokine panel has been described in our previously published paper (15). Reference values for cytokine release from healthy individuals were available for comparison.

### Outcomes

Primary outcomes were differences in immune cell populations and the induced immune response at admission between vaccinated and non-vaccinated patients hospitalized with severe COVID-19.

### Statistical analysis

Cases were matched by sex and age using the “nearest neighbor” matching method on propensity score (R statistical software). A ratio of 1:1 between cases and controls was used. Boxplots displaying medians and interquartile ranges and outliers (defined as outside 1.5 times the interquartile (IQ) upper and lower range) were used to visualize immune cell concentrations/proportions and induced cytokine release in cases and controls. Mann-Whitney U tests were used to compare the non-normally distributed continuous variables. Chi-sq and Fischer’s exact test assessed differences in categorical variables as appropriate. Adjustment for multiple testing was not conducted. This was decided because the study was exploratory, and the basis for hypothesis generation intended to identify potentially relevant variables for assessment in more detailed future studies. P-values <0.05 were considered significant. All statistical analyses were conducted using R statistical software (version 3.6.1) (22).

## Results

### Study population

A total of 20 patients were included in the study. The median age was 72 years (IQR 67–75) for non-vaccinated patients and 74 years (IQR 70 – 77) for vaccinated patients (*p=* 0.47). Seventy percent were men. No differences in comorbidities, BMI or immunocompromise were observed between vaccinated and non-vaccinated patients (**Table 1**). In addition, no differences in clinical outcomes, including mortality, ICU admission, or disease severity, were observed between vaccinated and non-vaccinated patients (**Table 2**). Two patients were infected with the Omicron variant, 13 with the Delta variant, and five patients had no available data regarding the variant. Regarding vaccinated patients, one received three doses at least 14 days before admission, and the remaining nine received two.

**Table 1:** Baseline Characteristics.

Variable	Overall, N = 20*^1^*	Vaccinated (cases), N = 10*^1^*	Non-vaccinated (controls), N = 10*^1^*	p-value*^2^*
Age	73 (69, 75)	74 (70, 77)	72 (67, 75)	0.47
Sex, Male	14 (70%)	7 (70%)	7 (70%)	>0.99
BMI	28 (26, 34)	29 (27, 34)	26 (26, 31)	0.53
Time since last SARS-CoV-2 vaccination (months)	6 (4, 9)	6 (4, 9)		
Comorbidities				
Any comorbidity	20 (100%)	10 (100%)	10 (100%)	
Cardiovascular	10 (50%)	5 (50%)	5 (50%)	>0.99
Isolated hypertension	12 (60%)	6 (60%)	6 (60%)	>0.99
Previous thromboembolic event	3 (15%)	1 (10%)	2 (20%)	>0.99
Hematologic	1 (5.0%)	1 (10%)	0 (0%)	>0.99
Neurologic	6 (30%)	3 (30%)	3 (30%)	>0.99
Psychiatric	4 (20%)	3 (30%)	1 (10%)	0.58
Pulmonary	6 (30%)	3 (30%)	3 (30%)	>0.99
Gastrointestinal	5 (25%)	2 (20%)	3 (30%)	>0.99
Hepatic	0 (0%)	0 (0%)	0 (0%)	
Diabetes	5 (25%)	3 (30%)	2 (20%)	>0.99
Renal	2 (10%)	2 (20%)	0 (0%)	0.47
Organ transplantation	0 (0%)	0 (0%)	0 (0%)	
Rheumatologic	3 (15%)	2 (20%)	1 (10%)	>0.99
Active cancer	1 (5.0%)	1 (10%)	0 (0%)	>0.99
Other comorbidity	18 (90%)	9 (90%)	9 (90%)	>0.99
Immunodeficiency	2 (10%)	2 (20%)	0 (0%)	0.47
**Cause of immunodeficiency**				>0.99
Etanercept treatment	1 (50%)	1 (50%)	0 (0%)	
Myelomatosis	1 (50%)	1 (50%)	0 (0%)	

Table describing baseline and clinical characteristics of all included patients. Regarding comorbidities, the table describes the number of patients (n) with any of the specified comorbidities in the table. “Other comorbidities” describes number of patients (n) with other comorbidities than the specified comorbidities in the table.

^1^ Data is described as medians (IQR) or n (%) depending on the datatype of the specific variable.

^2^ P-values were calculated using Mann-Whitney U test, Fisher's exact test or Pearson's Chi-squared test depending on the datatype of the specific variable.

**Table 2:** Clinical Characteristics.

Variable	Overall, N = 20*^1^*	Non-vaccinated, N = 10*^1^*	Vaccinated, N = 10*^1^*	*p*-value*^2^*
< 5L oxygen, n (%)	10 (50)	5 (50)	5 (50)	>0.99
> 5L oxygen, n (%)	1 (5.0)	1 (10)	0 (0)	>0.99
High flow oxygen therapy, n (%)	8 (40)	4 (40)	4 (40)	>0.99
NIV, n (%)	1 (5.0)	0 (0)	1 (10)	>0.99
Respirator, n (%)	0 (0)	0 (0)	0 (0)	
Early Warning Score (EWS) at admission, median (IQR)	5.00 (2.00 – 6.25)	5.00 (2.50 – 7.25)	5.00 (2.50 – 6.00)	0.85
**Disease Severity, n (%)**				>0.99
Low	11 (55)	6 (60)	5 (50)	
High	9 (45)	4 (40)	5 (50)	
Death during admission, n (%)	1 (5.0)	1 (10)	0 (0)	>0.99
Length of admission, Median (IQR)	4 (4 – 12)	6 (3 – 11)	4 (4 – 12)	0.97
Admission to ICU, n (%)	1 (5.0)	0 (0)	1 (10)	>0.99
Death within 90 days of admission, n (%)	2 (10)	1 (10)	1 (10)	>0.99

Table describing the clinical characteristics of all included patients during their admission. First available Early Warning Score (EWS) for each patient was used in the table.

NIV, non-invasive ventilation; ICU, intensive care unit.

^1^ Variables are described as medians (IQR) or n (%) depending on the datatype of the specific variable. ^2^ P-values were calculated using Mann-Whitney U test, Fisher's exact test or Pearson's Chi-squared test depending on the datatype and frequency of the specific variable.

### Immune cell profiles

**Figure 1:**
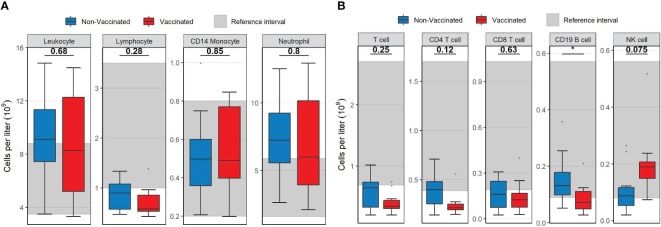
Boxplots visualizing the difference in the concentration of main leukocyte subsets between vaccinated (red) and non-vaccinated (blue) patients. **(A)** The difference in leukocytes, lymphocytes, CD14 monocytes and neutrophils. **(B)** The difference in T-cells, CD4 T-cells, CD8 T-cells, CD19 B-cells and NK-cells. Reference interval with data from healthy individuals are marked in grey in the background in both **(A, B)** P-values were calculated using Mann-Whitney U tests and displayed at the top of the boxplots.* P < 0.05. NK cell, natural killer cell. Mann-Whitney U test was used to assess differences between the groups.

Absolute concentrations of neutrophils and monocytes were similar for vaccinated and non-vaccinated patients. Vaccinated patients displayed overall lymphopenia (median 0,58x10^9^/L, IQR 0,326) compared to non-vaccinated patients who presented with a lymphocyte concentration in the lower end of the normal reference interval (median 0,898x10^9^/L, IQR 0,505, **Figure 1A**). Within the lymphocyte subpopulation, the absolute concentration of CD19 B-cells was significantly reduced in vaccinated patients compared to non-vaccinated patients (*p=* 0.035, **Figure 1B**). Additionally, the concentration of T-cells, in particular CD4 T-cells, was reduced in both groups compared to the normal reference interval. Though not significant, the median level and range of the CD4 T-cell count indicated a lower concentration in most vaccinated patients (*p=* 0.12, **Figure 1B**) compared to non-vaccinated patients. The CD8 T-cell counts were comparable in the two patient groups (p=0.63, **Figure 1B**) but with medians below the normal range. NK-cell concentration was within the normal range in both patient groups, but it was moderately elevated in vaccinated patients compared to non-vaccinated patients (*p=* 0.075, **Figure 1B**).

**Figure 2:**
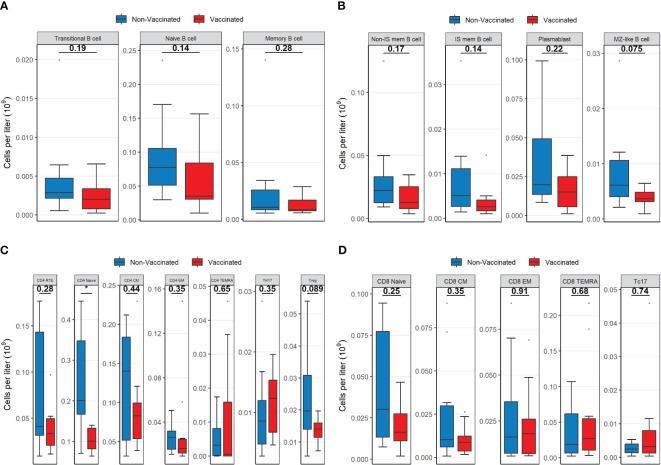
Boxplots visualizing the differences in the concentrations of B-cell lineages, CD4 lineages and CD8 lineages between vaccinated (red) and non-vaccinated (blue) patients. **(A)** The difference in B cell subsets. **(B)** The difference in B cell subsets. **(C)** The difference in CD4 subsets. **(D)** The difference in CD8 subsets. P-values were calculated using Mann-Whitney U tests and displayed at the top of each of the boxplots. * P < 0.05. Non-IS mem B cell, non-isotype switched memory B cell; IS mem B cell, isotype switched memory B cell; MZ-like B cell, marginal zone-like B cell; RTE, Recent thymic emigrants; CM, Central memory; EM, Effector memory; TEMRA, T effector memory CD45RA; TH17, T helper 17 cells; Treg, T regulatory cells.

The lower B-cell concentration in vaccinated patients was mainly caused by lower concentrations of naïve B-cells and isotype switch memory B-cells (**Figures 2A, B**). However, there were no significant differences in the distribution of B-cell subpopulations between the patient groups (**Supplementary Figure S1**).

Within the CD4 T-cell compartment, vaccinated patients displayed a significantly lower concentration of naïve T-cells compared to non-vaccinated patients (*p =* 0.015, **Figure 2C**). However, the concentrations of CD4 recent thymic emigrant T-cells (RTE) were not significantly different between the two patient groups (*p =* 0.28). Regulatory T- (Treg) cell concentrations were moderately reduced in vaccinated patients compared to non-vaccinated patients (**Figure 2C**). Neither of the two patient groups showed pronounced signs of immune activation (upregulation of HLA-DR) nor signs of exhaustion (CD57 and PD1 upregulation) within CD4 T-cells, and no significant differences were observed between the two groups (**Supplementary Figure S2A**).

Within CD8 T-cell populations, there were no differences in concentrations of subpopulations (**Figure 2D**). However, vaccinated patients had a significantly lower fraction of naïve CD8 T-cells compared to non-vaccinated patients (**Supplementary Figure S3**). Both patient groups showed similarly increased levels of immune activation (upregulation of HLA-DR) regarding CD8 T-cells, as well as similar signs of exhaustion (CD57 and PD1 upregulation). The check-point inhibitor (Tim3, CD366) expression on CD8 T-cells was not elevated in the two groups, although significantly higher in non-vaccinated patients than in vaccinated patients (*p =* 0.043, **Supplementary Figure S2B**).

**Figure 3:**
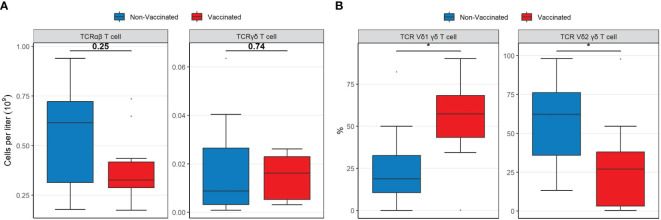
Boxplots visualizing the differences in the concentrations and percentages of TCR T cell types between vaccinated (red) and non-vaccinated (blue) patients. **(A)** The difference in TCRαβ and TCRγδ T cell concentrations. **(B)** The difference in TCRγδ1 and TCRγδ2 percentages. P-values were calculated using Mann-Whitney U tests and displayed at the top of each of the boxplots. * P < 0.05. TCR, T-cell receptor. Mann-Whitney U test was used to assess differences between the groups.

Finally, the overall concentrations of TCRγδ T-cells were comparable between the two patient groups (**Figure 3A**). However, vaccinated patients displayed a significantly higher proportion of TCRγδ1 T-cells (*p =* 0.019) and a lower proportion of TCR**γ**δ2 T-cells (*p =* 0.035) compared to non-vaccinated patients (**Figure 3B**). Median cell population concentrations and interquartile ranges of all patients according to their vaccination status are presented in **Supplementary Tables S1**, **S2**.

### TruCulture induced immune response

**Figure 4:**
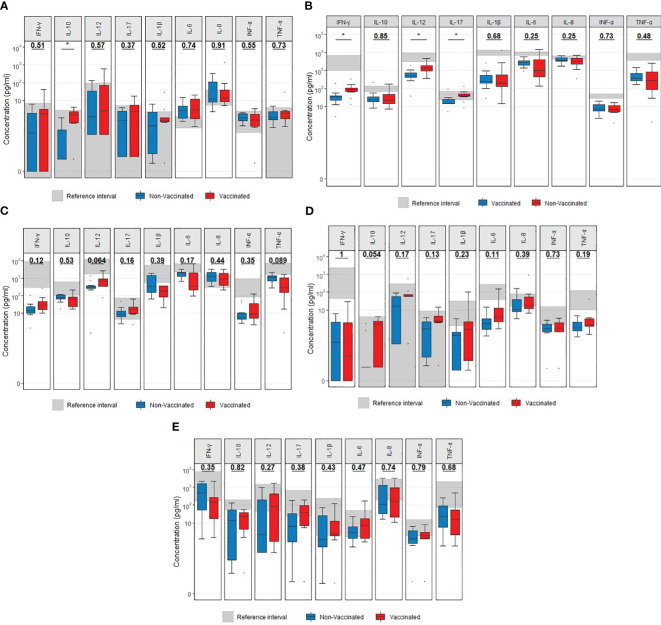
Boxplots visualizing the difference in cytokine concentrations at admission between vaccinated (red) and non-vaccinated (blue) patients. **(A)** Unstimulated. **(B)** LPS stimulation. **(C)** R848 stimulation. **(D)** PolyIC stimulation. **(E)** anti CD3/CD28 stimulation.P-values were calculated using Mann-Whitney U tests and displayed at the top of each of the boxplots. * p < 0.05. LPS, lipopolysaccharide; R848, Resiquimod; PolyIC, Polyinosinic:polycytidylic acid; CD3+CD28, Cluster of Differentiation 3 and 28.

Vaccinated patients displayed a significant increase in the unstimulated release of IL-10 (*p =* 0.015) compared to non-vaccinated patients (**Figure 4A**) and a higher LPS-induced release of IL-17A (*p =* 0.023), IL-12 (*p =* 0.011) and IFN-γ (*p* = 0.029) compared to non-vaccinated (controls) patients (**Figure 4B**). No other differences in the induced cytokine release between vaccinated and non-vaccinated patients were observed (R848, PolyIC and CD3/CD28, **Figures 4C-E**). To address the impact of observed outliers on the significant results in the presented boxplots with cytokine data, Mann Whitney U-tests were performed after the exclusion of outliers. Regarding the observed differences following LPS stimulation, following the exclusion of outliers, IFN-γ (*p=* 0.044), IL-12 (*p=* 0.006) and IL-17A (*p=* 0.001) remained significantly different. Regarding the unstimulated (NULL) results, IL-10 (*p=* 0.002) remained significantly different after excluding outliers.

## Discussion

This study compared immunologic signatures between vaccinated and non-vaccinated patients hospitalized with COVID-19. The main findings were that vaccinated patients (cases) admitted with with vaccine breakthrough COVID-19 infections had lower concentrations of CD19 B-cells and naïve CD4 T-cells and a higher proportion of TCRγδ1 T-cells. Furthermore, a higher unstimulated immune cell release of IL-10 at admission was observed.

We observed a lower B-cell concentration in vaccinated patients than in non-vaccinated patients admitted with COVID-19. It is well known that B-cell subsets, such as memory and plasma cells, are needed to protect from reinfection during repeated SARS-CoV-2 exposure (23). A reduced humoral response following vaccination could contribute to impaired COVID-19 immunity and hence admission to hospital despite COVID-19 vaccination. B-cell concentration has been strongly correlated with SARS-CoV-2 antibody production in immunocompromised patients (24). However, this association has not been observed in immunocompetent patients (25, 26). Our results, showing decreased B-cell concentrations in vaccinated patients, may indicate a suboptimal B-cell function in these patients, which would potentially contribute to hospital admission despite vaccination.

T-cells are crucial in antiviral immunity and are essential in immunological protection from SARS-CoV-2 infection (27). We observed T-cell cytopenia, particularly CD4 T-cells, and a lower concentration of naïve CD4 T-cells in vaccinated patients than in non-vaccinated patients. Several studies have reported that a successful response to vaccination is associated with a solid antigen-specific CD4 T-cell response (28–30). On the contrary, a low concentration of T-cells, including naïve CD4 T-cells, has been associated with poor vaccination responses. Some studies have also reported that a low concentration of naïve CD4 T-cells is associated with more severe COVID-19 (31–33). Our findings of a lower concentration of naïve CD4 T-cells in vaccinated patients may indicate a decreased T-cell repertoire and a smaller pool of T-cells to generate SARS-CoV-2 specific T-cells. This would ultimately increase the risk of impaired SARS-CoV-2 immunity. T- cells have been observed to be an important part of the immune response against SARS-CoV-2, where T-cell responses have a robust cross-recognition of SARS-CoV-2 variants despite varying antibody responses (34). This could suggest that vaccinated patients with severe COVID-19 due to breakthrough infection may have a suboptimal T-cell-mediated immune response, which should be further explored. CD4 T-cells are essential to achieve optimal B-cell function, and our finding of significantly reduced naïve CD4 T-cell counts may contribute to or may be related to reduced B-cell function. A study has observed a positive correlation between SARS-CoV-2 naïve CD4 T cells and viral clearance, where viral clearance increased with increasing SARS-CoV-2 specific naïve CD4 T cells (35). The authors of this study suggest, in concordance with the conclusion of our T-cell findings, that low counts of SARS-CoV-2 specific naïve CD4 T cells could implicate an insufficient B-cell activation leading to a decreased viral clearance (35). We suggest that an insufficient B-cell activation due to a suboptimal T-cell response could potentially affect the overall humoral response, leading to SARS-CoV-2 breakthrough infections. However, the measurement of SARS-CoV-2 specific T-cells combined with an assessment of the indirect effect on B-cell activation is required to confirm this.

Most studies have focused on classical TCRαβ T-cells when describing the role of T-cells in COVID-19. In contrast, few studies have explored the role of TCRγδ T-cells in COVID-19 (36, 37). TCRγδ1 and TCRγδ2 are the two main TCRγδ subsets and the most studied. TCRγδ T-cells, a component with innate-like immune system properties, respond to inflammation and stressed or infected cells (38). *In vitro*, TCRγδ T-cells have also shown the ability to eliminate SARS-CoV-2 (39). TCRγδ T-cells are therefore considered an essential part of the innate defense against viruses, including SARS-CoV-2. TCRγδ2 T-cells can interact with other immune cells, including B-cells and dendritic cells, and carry out cytolysis through the effects of cytotoxic perforin and granzymes (40, 41).

Furthermore, a recent study observed an association between TCRγδ T-cells and disease severity, where a low frequency of TCRγδ2 T-cells, which usually are the dominant TCRγδ T-cells in adults, were associated with severe COVID-19 disease (42–44). In our study, we observed a markedly higher proportion of TCRγδ1 T-cells and a lower proportion of TCRγδ2 T-cells in vaccinated patients compared to non-vaccinated patients. Overall, these observations could indicate a suboptimal TCRγδ T-cell response in vaccinated patients, where previous studies have shown that TCRγδ T-cells are highly involved and play an essential role in the host immune response against SARS-CoV-2 (38). Our findings could indicate that the difference in TCRγδ T-cell proportions in vaccinated patients could contribute to a non-favorable immune cell profile, potentially leading to subsequent hospital admission. These findings may further underline the importance of T-cells during SARS-CoV-2 infection.

We assessed the *ex vivo*-induced immune response by TruCulture as a proxy for immune function. We observed a significant increase in the unstimulated release of IL-10 in vaccinated patients. This observation may reflect both high unstimulated *ex vivo* release of IL-10 during incubation and high circulating plasma levels of IL-10. IL-10 is a classical anti-inflammatory cytokine with immunosuppressive activities, downregulating various cytokines and co-stimulatory molecules (45). Several studies have reported that early production of IL-10 is associated with poor clinical outcomes in COVID-19 (46–48). A recent meta-analysis further concluded that circulating IL-10 can be used as a predictor for patients’ clinical status and survival, where IL-10 is suggested to be the main cause of the immunodepression associated with SARS-CoV-2 infection (49). However, further studies with larger study populations are needed to confirm our finding of higher *ex vivo* release of IL-10/higher circulating levels of IL-10 in patients with SARS-CoV-2 vaccine breakthrough infection and to determine the potential influence of this phenomenon on the COVID-19 disease course.

Research on IL-10 in the context of COVID-19 has thoroughly evaluated its role, revealing that higher levels of IL-10 correlate with more severe disease and have been shown to predict the progression to severe or critical disease (47, 48). These studies were larger and focused on the clinical impact of IL-10, rather than the immunological phenotype of severe COVID-19 stratified by vaccination status. In this study, we did not observe a difference in disease severity between vaccinated and non-vaccinated patients. The study’s aim was exploratory regarding immunological phenotype, and our findings should, therefore, be interpreted as indicative rather than conclusive. Further studies with a larger sample size assessing SARS-CoV-2 specific immune cells, antibodies, the effect of clinical variables on observed immune response differences and samples taken before COVID-19 (or a follow-up sample) are highly warranted to fully evaluate the underlying pathophysiology and potential causes regarding our observed immune response differences.

Our study has strengths and limitations. First, we present detailed, in-depth immunological profiles of patients hospitalized with severe COVID-19 at admission, providing valuable insights into the immune responses of vaccinated and non-vaccinated individuals otherwise underreported in the literature (50, 51). In contrast to other reports (48, 49), we did not only focus on specific immune cells but had a rather broad approach with data on cytokine profiles, B cells, T cells, and T cell receptor profiles, activation, and exhaustion. This very deep and broad immunophenotyping, paired with data on inflammation through cytokine assessment, is unique and scarce in the literature. We could not find similar studies regarding deep and broad immunophenotyping combined with cytokine responses stratified by vaccination status in patients hospitalized with severe COVID-19. We applied strict inclusion criteria, ensuring a well-defined study population and minimizing the potential risk of confounding. The observational nested case-control design facilitates comparing immunological characteristics between different patient groups, contributing to a better understanding of vaccine breakthrough infections.

We acknowledge that the results of our study are limited by a relatively small sample size, emphasizing the need for more extensive studies to validate findings. We consider this study a descriptive, hypothesis-generating study, with its strength in deep immunophenotyping and broad immunological analysis, coupled with strict sex and age case matching. Also, only patients with community-acquired SARS-CoV-2 infections were included. Thus, we aimed to optimize the comparison between vaccinated vs. non-vaccinated groups, which constituted the premise of our study. Despite our efforts, recruiting sex- and age-matched non-vaccinated patients hospitalized with COVID-19 was challenging, likely because of Denmark’s widespread acceptance of COVID-19 vaccination (52). This contributed, at least in part, to the limitations in the sample size. We also included two immunocompromised individuals in the cohort. Even though the number of individuals is minimal and no conclusions can be drawn, the detailed description is of value since this group of patients is likely to constitute a large majority of patients with breakthrough and persistent SARS-CoV-2 infections.

Finally, adding a third comparison group with vaccinated, non-hospitalized patients and antibody titers would have been ideal. Still, previous studies, including by our group, assessed neutralizing antibody titers for up to 6 months following primary COVID-19 infection. Although many patients may still have high neutralizing antibody titers (18), differences in antibody persistency and response to vaccination may contribute to the occurrence of breakthrough infections. The absence of SARS-CoV-2-specific immune cell stimulations and antibody measurements also limits our study. Furthermore, samples were taken during severe disease, challenging the interpretation of the results. In the future, studies should aim to overcome these challenges by, for instance, allowing the recruitment across multiple sites, including different groups of immunocompromised patients, thereby aiming for generalizability and robustness of findings.

## Conclusions

Overall, the differences observed in this study between vaccinated and non-vaccinated patients admitted to hospitals with COVID-19 suggest that vaccinated patients display a non-favorable immune response, which previous studies have identified as potentially associated with immune impairment. We observed low total B-cell counts, low CD4 naïve T-cells, a skewed TCRγδ V1/V2 ratio, and an exaggerated IL-10 response in vaccinated compared to non-vaccinated patients. These observations align with previous studies, where similar findings in B-cells, T-cells, and release of/circulating IL-10 have been associated with a weak response to vaccination and more severe clinical disease (23, 31–33, 42, 48, 49, 53). We suggest a potentially suboptimal response to vaccination, perhaps due to an underlying degree of immune impairment, could contribute to the observed differences. However, further studies, including simultaneous serological analyses, are required to assess this. Continued research and further follow-up studies evaluating these immunological differences are warranted.

## Data availability statement

The datasets presented in this article are not readily available because data can be made available by the investigators after review of a suitable protocol by the steering committee of the study group upon request. Requests to access the datasets should be directed to Sisse.Rye.Ostrowski@regionh.dk; zitta.barrella.harboe@regionh.dk; Hanne.Marquart@regionh.dk.

## Ethics statement

The study was approved by the Danish Ethics Committee (H-20026502) and the Danish Data Protection Agency (P-2020-426). The studies were conducted in accordance with the local legislation and institutional requirements. The participants provided their written informed consent to participate in this study.

## Author contributions

AS: Conceptualization, Data curation, Formal Analysis, Funding acquisition, Investigation, Methodology, Project administration, Writing – original draft, Writing – review & editing. HH: Conceptualization, Data curation, Formal Analysis, Investigation, Methodology, Project administration, Supervision, Writing – review & editing. JH: Supervision, Writing – review & editing. LK: Investigation, Project administration, Writing – review & editing. BL: Funding acquisition, Investigation, Resources, Supervision, Writing – review & editing. AD: Investigation, Supervision, Writing – review & editing. FG: Investigation, Project administration, Writing – review & editing. MM: Investigation, Methodology, Project administration, Writing – review & editing. RT: Investigation, Methodology, Project administration, Writing – review & editing. CN: Conceptualization, Funding acquisition, Investigation, Project administration, Resources, Supervision, Writing – review & editing. PB: Investigation, Project administration, Writing – review & editing. CJ: Conceptualization, Supervision, Writing – review & editing. KF: Conceptualization, Funding acquisition, Project administration, Resources, Supervision, Writing – review & editing. TF: Conceptualization, Resources, Supervision, Writing – review & editing. HM: Conceptualization, Formal Analysis, Funding acquisition, Investigation, Methodology, Project administration, Resources, Software, Supervision, Writing – review & editing. ZH: Conceptualization, Funding acquisition, Investigation, Methodology, Project administration, Resources, Supervision, Writing – original draft, Writing – review & editing. SO: Conceptualization, Formal Analysis, Funding acquisition, Investigation, Methodology, Project administration, Resources, Supervision, Writing – review & editing.
