# Supplementary material for: Immune cell populations and induced immune responses at admission in patients hospitalized with vaccine breakthrough SARS-CoV-2 infections
Source: Front Immunol. 2024 Jun 5;15:1360843. doi: 10.3389/fimmu.2024.1360843 (PMC11188326; doi:10.3389/fimmu.2024.1360843)
Supplement: Supplementary file 1 [file Image_1.pdf]

Supplementary Figure 1 - B-cell lineage percentages

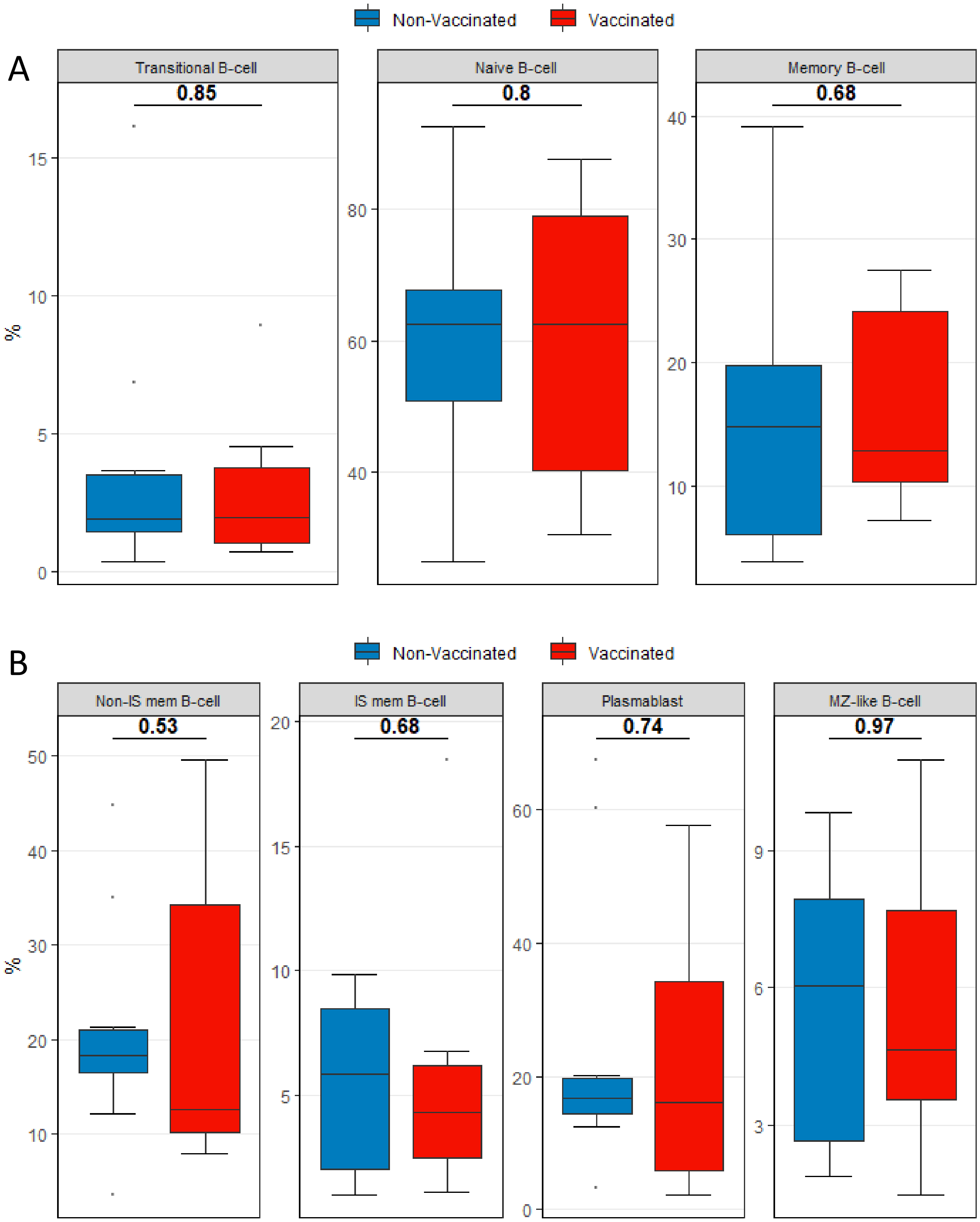

Supplementary Figure 1. Boxplots visualizing the difference in the proportions (%) of B cell lineages between vaccinated (red) and non-vaccinated (blue) patients. **A)** The difference in transitional B cells, naïve B cells and memory B cells. **B)** The difference in Non-isotype switch memory B cells, isotype switch memory B cells, plasmablasts and MZ-like B cells.
P-values were calculated using Mann-Whitney U tests and displayed at the top of the boxplots.
* $P < 0.05$
