# Supplementary material for: Immune cell populations and induced immune responses at admission in patients hospitalized with vaccine breakthrough SARS-CoV-2 infections
Source: Front Immunol. 2024 Jun 5;15:1360843. doi: 10.3389/fimmu.2024.1360843 (PMC11188326; doi:10.3389/fimmu.2024.1360843)
Supplement: Supplementary file 2 [file Image_2.pdf]

Supplementary Figure 2 - CD4/CD8 T-cell activation/exhaustion markers percentages

A

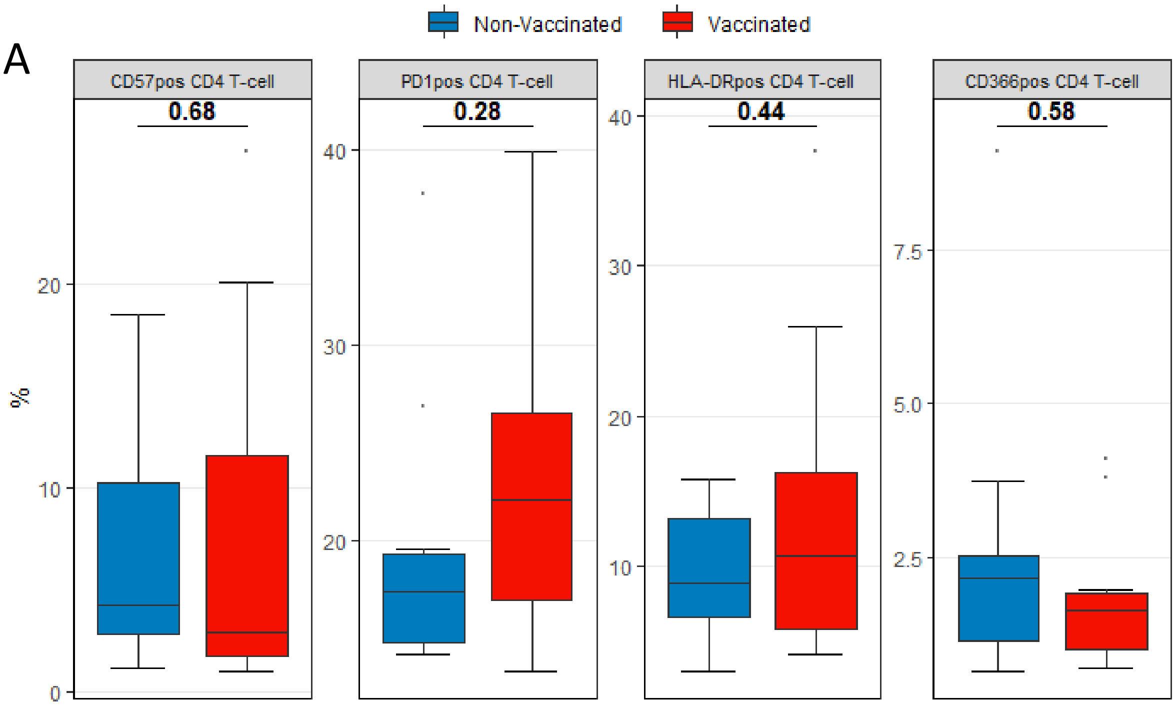

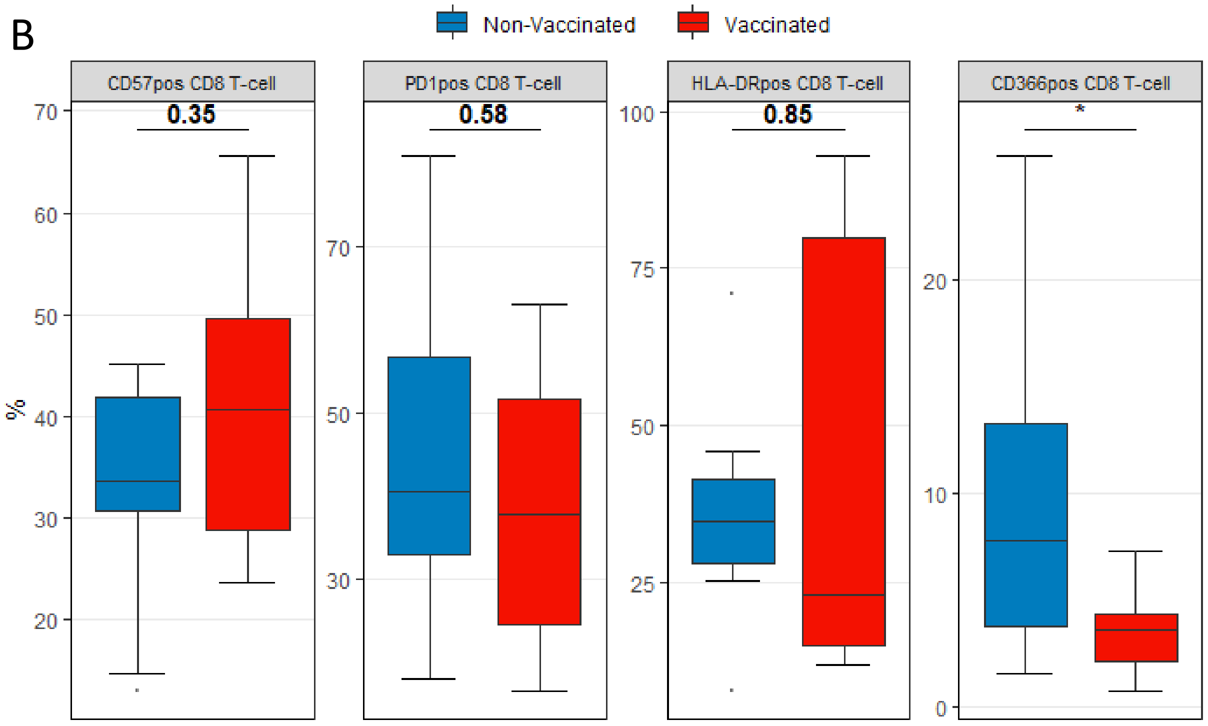

Supplementary Figure 2. Boxplots visualizing the difference in the proportions (%) of activation/exhaustion markers on CD4+ and CD8+ T cells between vaccinated (red) and non-vaccinated (blue) patients. **A)** The difference activation/exhaustion markers on CD4+ T cells. **B)** The difference activation/exhaustion markers on CD8 + cells.
P-values were calculated using Mann-Whitney U tests and displayed at the top of the boxplots.
* P < 0.05
