# Supplementary material for: Immune cell populations and induced immune responses at admission in patients hospitalized with vaccine breakthrough SARS-CoV-2 infections
Source: Front Immunol. 2024 Jun 5;15:1360843. doi: 10.3389/fimmu.2024.1360843 (PMC11188326; doi:10.3389/fimmu.2024.1360843)
Supplement: Supplementary file 4 [file Image_4.pdf]

Supplementary Figure 4 - CD4 lineage Percentages

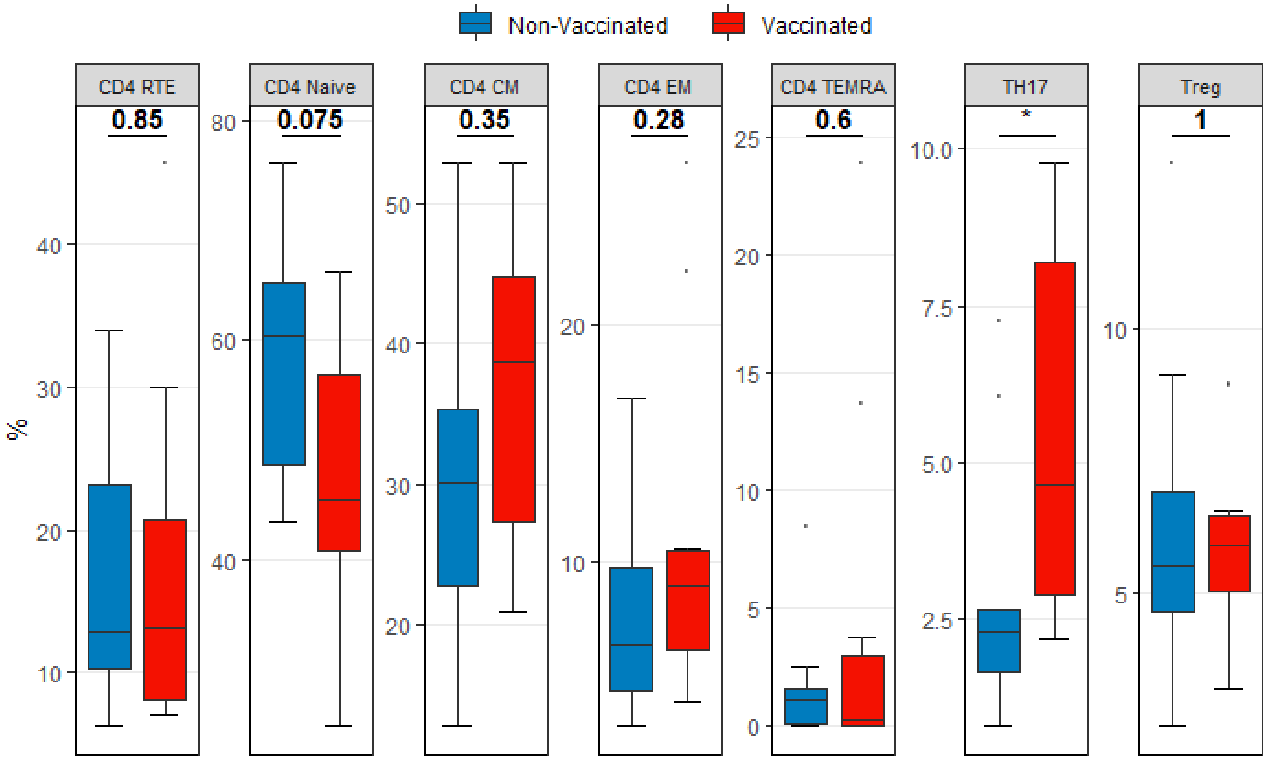

Supplementary Figure 4. Boxplots visualizing the difference in the proportions (%) of CD4+ subsets between vaccinated (red) and non-vaccinated (blue) patients.
P-values were calculated using Mann-Whitney U tests and displayed at the top of the boxplots.
* P < 0.05
