# Supplementary material for: Immune cell populations and induced immune responses at admission in patients hospitalized with vaccine breakthrough SARS-CoV-2 infections
Source: Front Immunol. 2024 Jun 5;15:1360843. doi: 10.3389/fimmu.2024.1360843 (PMC11188326; doi:10.3389/fimmu.2024.1360843)
Supplement: Supplementary file 5 [file Table_1.docx]

**Supplementary Table 1: Summary of cell population concentrations by vaccination status**

| Cell population | Median (10^9^/L), Non-Vaccinated | Median (10^9^/L), Vaccinated | IQR (10^9^/L), Non-Vaccinated | IQR (10^9^/L), Vaccinated |
| --- | --- | --- | --- | --- |
| Leukocyte | 9,123 | 8,273 | 3,903 | 7,063 |
| Lymphocyte | 0,898 | 0,578 | 0,505 | 0,326 |
| CD14 Monocyte | 0,498 | 0,490 | 0,241 | 0,373 |
| Neutrophil | 7,227 | 5,990 | 3,679 | 6,217 |
| T cell | 0,644 | 0,343 | 0,409 | 0,136 |
| CD4 T cell | 0,401 | 0,216 | 0,227 | 0,057 |
| CD8 T cell | 0,160 | 0,125 | 0,171 | 0,091 |
| CD19 B cell | 0,131 | 0,072 | 0,080 | 0,061 |
| NK cell | 0,087 | 0,190 | 0,063 | 0,058 |
| Transitional B cell | 0,003 | 0,002 | 0,003 | 0,003 |
| Naive B cell | 0,077 | 0,035 | 0,054 | 0,053 |
| Memory B cell | 0,011 | 0,008 | 0,017 | 0,010 |
| Non-isotype switch mem B cell | 0,022 | 0,013 | 0,021 | 0,018 |
| Isotype switch mem B cell | 0,005 | 0,003 | 0,008 | 0,002 |
| Plasmablast | 0,020 | 0,015 | 0,036 | 0,019 |
| MZ-like B cell | 0,006 | 0,004 | 0,007 | 0,002 |
| CD4 RTE | 0,041 | 0,033 | 0,112 | 0,029 |
| CD4 Naive | 0,200 | 0,101 | 0,181 | 0,051 |
| CD4 CM | 0,140 | 0,083 | 0,132 | 0,046 |
| CD4 EM | 0,026 | 0,016 | 0,016 | 0,012 |
| CD4 TEMRA | 0,003 | 0,000 | 0,008 | 0,016 |
| TH17 | 0,009 | 0,012 | 0,007 | 0,009 |
| Treg | 0,020 | 0,014 | 0,017 | 0,004 |
| CD8 Naive | 0,030 | 0,016 | 0,064 | 0,016 |
| CD8 CM | 0,010 | 0,009 | 0,024 | 0,009 |
| CD8 EM | 0,016 | 0,018 | 0,028 | 0,019 |
| CD8 TEMRA | 0,019 | 0,027 | 0,055 | 0,044 |
| Tc17 | 0,002 | 0,003 | 0,003 | 0,007 |
| TCRαβ T cell | 0,615 | 0,326 | 0,408 | 0,130 |
| TCRγδ T cell | 0,009 | 0,016 | 0,023 | 0,018 |

**Supplementary Table 1**. Overview over median cell population concentrations (10^9^/L) and interquartile range (IQR) stratified by vaccination status.
