# Supplementary material for: Immune cell populations and induced immune responses at admission in patients hospitalized with vaccine breakthrough SARS-CoV-2 infections
Source: Front Immunol. 2024 Jun 5;15:1360843. doi: 10.3389/fimmu.2024.1360843 (PMC11188326; doi:10.3389/fimmu.2024.1360843)
Supplement: Supplementary file 6 [file Table_2.docx]

**Supplementary Table 2: Summary of cell population percentages by vaccination status**

| Cell Population | Median (%), Non-Vaccinated | Median (%), Vaccinated | IQR (%), Non-Vaccinated | IQR (%), Vaccinated |
| --- | --- | --- | --- | --- |
| TCR Vδ1 γδ T cell | 18,74 | 57,27 | 22,06 | 24,91 |
| TCR Vδ2 γδ T cell | 62,27 | 27,00 | 40,28 | 34,85 |
| Transitional B cell | 1,89 | 1,96 | 2,09 | 2,72 |
| Naive B cell | 62,48 | 62,57 | 16,66 | 38,71 |
| Memory B cell | 14,77 | 12,86 | 13,73 | 13,83 |
| Non-isotype switch mem B cell | 18,33 | 12,58 | 4,59 | 24,12 |
| Isotype switch mem B cell | 5,85 | 4,28 | 6,49 | 3,76 |
| Plasmablast | 16,72 | 16,06 | 5,48 | 28,37 |
| MZ-like B cell | 6,06 | 4,66 | 5,28 | 4,11 |
| CD57pos CD4 T cell | 4,26 | 2,89 | 7,45 | 9,88 |
| PD1pos CD4 T cell | 17,42 | 22,11 | 4,51 | 9,50 |
| HLA-DRpos CD4 T cell | 8,92 | 10,69 | 6,50 | 10,42 |
| CD366pos CD4 T cell | 2,16 | 1,65 | 1,37 | 0,92 |
| CD57pos CD8 T cell | 33,64 | 40,65 | 11,21 | 20,85 |
| PD1pos CD8 T cell | 40,56 | 37,70 | 23,76 | 27,08 |
| HLA-DRpos CD8 T cell | 34,66 | 22,87 | 13,43 | 64,81 |
| CD366pos CD8 T cell | 7,86 | 3,65 | 9,46 | 2,23 |
| CD8 Naive | 29,60 | 13,63 | 13,81 | 9,31 |
| CD8 CM | 9,09 | 6,93 | 7,83 | 5,43 |
| CD8 EM | 16,03 | 16,30 | 12,55 | 20,23 |
| CD8 TEMRA | 23,04 | 29,91 | 17,15 | 31,04 |
| Tc17 | 1,50 | 2,84 | 0,36 | 3,74 |
| CD4 RTE | 12,91 | 13,12 | 12,96 | 12,59 |
| CD4 Naive | 60,40 | 45,44 | 16,61 | 16,20 |
| CD4 CM | 30,09 | 38,71 | 12,49 | 17,39 |
| CD4 EM | 6,58 | 9,01 | 5,19 | 4,16 |
| CD4 TEMRA | 1,09 | 0,22 | 1,49 | 2,92 |
| TH17 | 2,29 | 4,64 | 1,00 | 5,30 |
| Treg | 5,52 | 5,90 | 2,28 | 1,40 |

**Supplementary Table 2**. Overview over median cell population percentages of parent gate and interquartile range (IQR) stratified by vaccination status.
